# Improving the aesthetic outcome with burr hole cover placement in chronic subdural hematoma evacuation—a retrospective pilot study

**DOI:** 10.1007/s00701-018-3659-9

**Published:** 2018-08-28

**Authors:** Flavio Vasella, Kevin Akeret, Nicolas R. Smoll, Menno R. Germans, Elisabeth Jehli, Oliver Bozinov, Luca Regli, Martin N. Stienen, on behalf of the CORRECT SCAR study group, David Bellut, Sandra Dias, Giuseppe Esposito, Jorn Fierstra, Dilek Könü-Leblebicioglu, Niklaus Krayenbühl, Nicolai Maldaner, Marian C. Neidert, Markus Oertel, Carlo Serra, Lennart H. Stieglitz, Julia Velz, Jan-Karl Burkhardt

**Affiliations:** 1 Department of Neurosurgery, University Hospital Zurich, Zurich, Switzerland; 2 School of Population and Global Health, University of Melbourne, Melbourne, Australia; 3 Clinical Neuroscience Center, University of Zurich, Frauenklinikstrasse 10, 8091 Zurich, Switzerland

**Keywords:** Burr hole cover, Chronic subdural hematoma, Trepanation, Aesthetic outcome, Complications, Scar, Patient satisfaction, Burr hole plate

## Abstract

**Background:**

The aesthetic outcome after burr hole trepanation for the evacuation of chronic subdural hematomas (cSDH) is often unsatisfactory, as the bony skull defects may cause visible skin depressions. The purpose of this study was to evaluate the efficacy of burr hole cover placement to improve the aesthetic outcome.

**Methods:**

We reviewed consecutive patients treated by burr hole trepanation for cSDH with or without placement of burr hole covers by a single surgeon between October 2016 and May 2018. The clinical data, including complications, were derived from the institution’s prospective patient registry. The primary endpoint was the aesthetic outcome, as perceived by patients on the aesthetic numeric analog (ANA) scale, assessed by means of a standardized telephone interview. Secondary endpoints were skin depression rates and wound pain, as well as complications.

**Results:**

From *n* = 33, outcome evaluation was possible in *n* = 28 patients (*n* = 24 male; mean age of 70.4 ± 16.1 years) with uni- (*n* = 20) or bilateral cSDH (*n* = 8). A total of 14 burr hole covers were placed in 11 patients and compared to 50 burr holes that were not covered. Patient satisfaction with the aesthetic outcome was significantly better for covered burr holes (mean ANA 9.3 ± 0.74 vs. 7.9 ± 1.0; *p* < 0.001). Skin depressions occurred over 7% (*n* = 1/14) of covered and over 92% (*n* = 46/50) of uncovered burr holes (*p* < 0.001). There was no difference in wound pain (*p* = 0.903) between covered and uncovered sites. No surgical site infection, cSDH recurrence, or material failure was encountered in patients who had received a burr hole plate.

**Conclusions:**

In this retrospective series, placement of burr hole covers was associated with improved aesthetic outcome, likely due to reduction of skin depressions. A randomized controlled trial is developed to investigate whether adding burr hole covers results in superior aesthetic outcomes, without increasing the risk for complications.

## Introduction

Chronic subdural hematoma (cSDH) is one of the most common conditions encountered in the neurosurgical care of elderly patients. Due to demographic changes as well as widespread use of antiplatelet and anticoagulant agents, its incidence is further rising [8]. While the roles of adjuvant and multidisciplinary treatment are under evaluation, surgical evacuation remains the backbone in the management of patients with cSDH [1, 3, 8].

Burr hole trepanation for cSDH is a minimally invasive procedure, allowing for rapid relief of space-occupying hematomas and clinical improvement. The likelihood for recovery of neurological deficits or general functional disability is high (80–90%) in otherwise healthy patients [2, 9]. Today, there is little doubt that burr hole trepanation is both safe and effective for this indication.

**Fig. 1:**
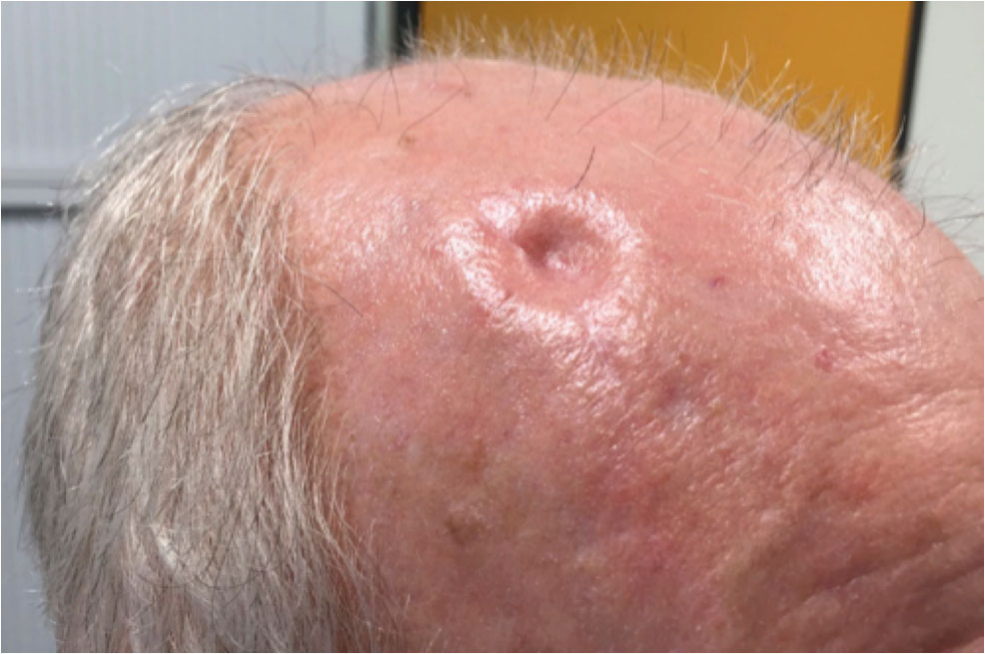
Illustrative picture of a 76-year-old male patient, seen in outpatient clinics about 12 weeks after right-sided frontal and parietal burr hole trepanation for a cSDH, with the trepanation site not covered by a burr hole plate

One downside of burr hole trepanation, however, is the unsatisfactory aesthetic outcome, as the skin just above the trepanation site frequently sinks in after hematoma reabsorption (Fig. 1). Some patients feel distinctly troubled by these stigmatizing and often well-visible skin depressions, and it may even interfere with activities of daily life (ADLs) [7]. In theory, placing a burr hole cover after hematoma evacuation may prevent from this undesirable cosmetic defect. This minor deviation from the regular surgical procedure has been performed by many neurosurgeons over the last decades but is poorly studied and has not yet found entry into clinical practice as standard of care.

## Methods and materials

The aim of this retrospective study was to evaluate whether the placement of a burr hole cover after burr hole trepanation for cSDH improves patient satisfaction with the aesthetic result. For this, we reviewed the institutional patient registry [13] to identify consecutive patients who underwent burr hole trepanation for the evacuation of uni- or bilateral cSDH by a single surgeon (M.N.S.) between October 2016 and May 2018. The single-surgeon approach was chosen, as most other faculty neurosurgeons do not or only rarely place burr hole covers for this indication.

Clinical and complication data were withdrawn from the department’s prospective database of complications and outcome, and disease-specific data were added by reviewing electronic patient records [9, 13].

### Placement of burr hole covers

Depending on the surgeon’s preference, one titanium plate (Stryker® UN3 BURR HOLE COVER, 20 mm, W/TAB, item code 53-34520) was placed per burr hole, fixed with two screws (Stryker® UNIII AXS SCREWS, SELF-DRILLING, 1.5 × 4MM, item code 56-15934) in some patients. There were no factors that were strictly and systematically associated with burr hole cover placement, but the surgeon was more likely to choose bald patients and patients in otherwise good functional condition (active participation in life) to receive a burr hole cover. In general, the frontal burr hole was preferably covered while the parietal burr hole was more often left uncovered, especially in patients with scalp hair. No burr hole plates were placed in patients with known systemic infections or allergic reaction against titanium. Anticoagulation or antiplatelet medication was no exclusion criterion for adding a burr hole cover. Patients having received a burr hole cover were informed about the device placement.

### Standard management of patients with cSDH

In our department, double burr hole trepanation (14 mm trepan) is typically performed under general anesthesia with the patient placed in supine position and rotated head on a ring-shaped gel cushion [9, 10]. We place the frontal burr hole at the junction of the superior temporal line and the coronal suture (stephanion), the posterior burr hole in the region of the parietal eminence. After trepanation and dural opening, we evacuate the hematoma by irrigation with warmed saline solution. We consider drain placement as standard of care [12] and prefer subperiosteal over subdural drain placement for its better safety profile [2, 15]. When the burr hole cover is placed, it is securely pressed onto the skull and fixed with two screws. In order to prevent screws from falling into the subdural space, it is imperative that the plate covers the burr hole completely before the screw is received from the scrub nurse. Postoperatively, patients remain immobilized and flat in supine position for 48 h until the drain is removed.

### Outcome assessment

During a standardized telephone interview (in June 2018) by a physician not involved in the primary treatment, patients were asked to separately rate the following items per burr hole:

- Satisfaction with the aesthetic result of the scar using the aesthetic numeric analog (ANA) scale, ranging from 0 (unsatisfied) to 10 (very satisfied) [4]
- Subjective impression whether or not the skin is depressed (yes or no)
- Wound pain measured on the numeric rating scale (NRS); ranging from 0 (no pain) to 100 (extreme pain)

In addition, we recorded the rate of intra- and typical postoperative complications per patient, including surgical site infections (SSIs) and recurrent cSDH requiring reoperation, but also material failure.

### Statistical considerations

The null hypothesis was that the satisfaction with the aesthetic result of the operation does not differ between burr holes that are covered (intervention) or not covered by a burr hole plate (control). The primary endpoint was the aesthetic outcome on the ANA scale. Secondary endpoints were the rates of skin depression and complications, as well as wound pain.

For analysis of the primary endpoint, the results obtained in the intervention group were compared to those of the control group, reported per burr hole. The same applies to the analysis of skin depression and wound pain. As the remaining secondary outcomes were not burr hole-specific but reflected the patient condition as a whole, the remaining secondary endpoints were reported on a per-patient basis.

For the primary endpoint (quantitative variable on an interval scale), a rank-sum test was performed to analyze group differences. The same was applied for analysis of the NRS-pain outcome, as results were not normally distributed. The rates of skin depression and complications were analyzed using Fisher’s exact tests.

### Ethical considerations

The local ethics committee (Kantonale Ethikkommission KEK-ZH 2012–0244) approved the prospective collection and analysis of data in the patient registry. The study was registered at http://clinicaltrials.gov (NCT01628406) and follows the STROBE recommendation for observational studies. All patients consented to the additional telephone interview. No funding was received for this study. The authors report no conflicts of interest.

## Results

A total of *n* = 33 patients were identified, of which *n* = 5 patients had to be excluded for study purpose (*n* = 2 deceased, unrelated to the surgical intervention; *n* = 2 patients lost to follow-up despite all efforts; *n* = 1 patient reached, but unable to speak). None of these five excluded patients had received a burr hole cover.

**Fig. 2:**
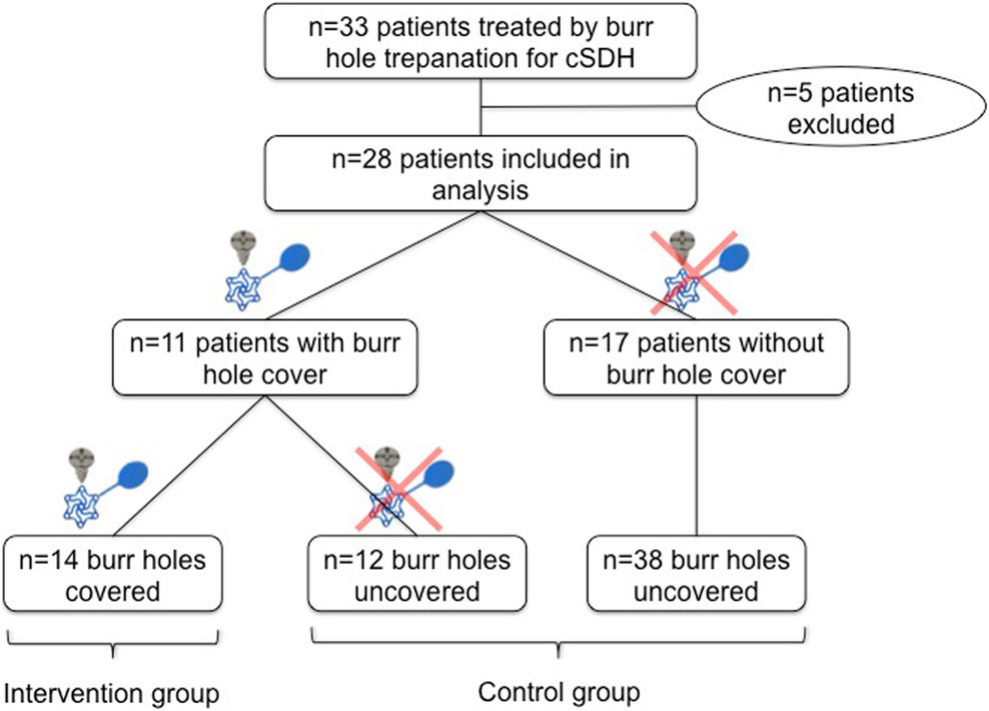
Study algorithm demonstrating how the intervention and control groups were arrived at

The remaining 28 patients (*n* = 24 male; 85.7%) had a mean age of 70.4 (± 16.1 years, standard deviation (SD)) and uni- or bilateral cSDH in 20 (71.4%) or 8 cases (28.6%), respectively (Table 1). A total of 14 burr hole plates (intervention group) were placed in 11 patients (39.3%), 1 single burr hole plate in *n* = 8, and 2 burr hole plates in *n* = 3. A total of 50 burr holes were not covered by plates (control group). A study algorithm can be found in Fig. 2.

**Table 1:** Characteristics of included patients, as well as site and number of applied burr hole covers. *CSDH* chronic subdural hematoma

No.	Sex	Age	Side of cSDH	Burr hole cover
1	Female	76	Both	No
2	Male	77	Left	No
3	Female	52	Left	No
4	Male	92	Both	No
5	Male	80	Left	No
6	Male	77	Left	No
7	Male	83	Left	No
8	Male	50	Left	No
9	Male	49	Right	No
10	Male	52	Left	Yes (1; parietal left)
11	Male	64	Right	No
12	Male	93	Left	No
13	Male	70	Left	Yes (1; frontal left)
14	Male	87	Right	No
15	Male	76	Right	Yes (1; frontal right)
16	Male	35	Right	No
17	Male	62	Both	Yes (2; frontal and parietal left)
18	Male	78	Both	Yes (1; frontal right)
19	Male	91	Both	Yes (1; frontal right)
20	Male	30	Left	No
21	Male	69	Left	Yes (1; frontal left)
22	Male	72	Both	Yes (2; frontal left and frontal right)
23	Male	74	Right	Yes (1; frontal right)
24	Male	73	Both	No
25	Male	76	Both	Yes (2; frontal and parietal left)
26	Female	86	Left	No
27	Female	62	Right	No
28	Male	71	Both	Yes (1; frontal left)

### Analysis of the primary endpoint

**Fig. 3:**
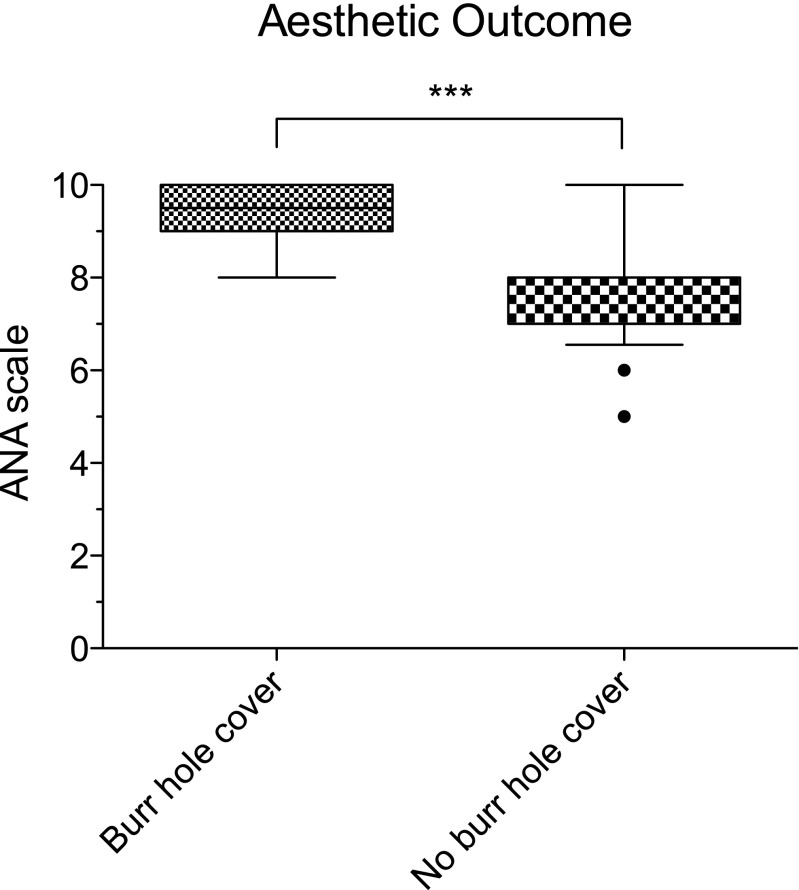
Box plot (25th to 75th percentile and mean) with whiskers (5th–95th percentile) and outliers (points). The mean aesthetic satisfaction (ANA) score in the intervention group was 9.3 (SD 0.74) and 7.9 in the control group (SD 1.0, ***p < 0.001)

The mean ANA score in the intervention group was 9.3 (SD 0.74) and 7.9 in the control group (SD 1.0, *p* < 0.001; Fig. 3).

### Analysis of the secondary endpoints

**Fig. 4:**
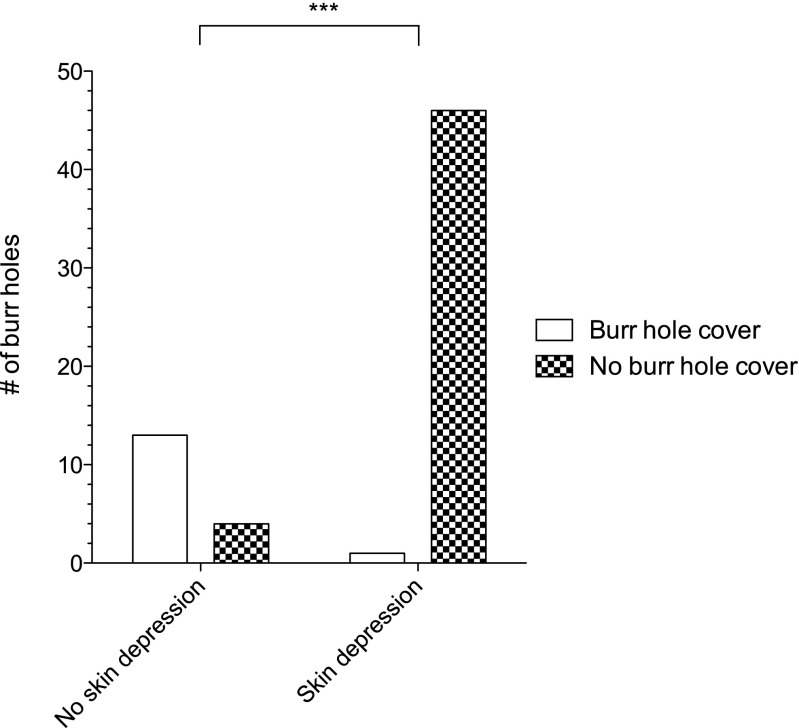
The rate of skin depressions over covered burr holes was 7% (1/14) and 92% (46/50) over burr holes that are not covered (***p < 0.001)

The rate of skin depressions over covered burr holes was 7% (1/14) in the intervention and 92% (46/50) in the control group (*p* < 0.001; Fig. 4), respectively. Mean NRS wound pain was 1.4 (SD 5.3) in the intervention and 2.8 (SD 10.5) in the control group (*p* = 0.903).

The cohort was followed for a mean of 270 days (SD 188), with a longer mean follow-up in patients with a burr hole cover (346 days, SD 193) versus those without a burr hole cover (161 days, SD 115; *p* = 0.019). There was no intraoperative complication associated with burr hole cover placement. There was no SSI and no material failure in the series. One patient, who had not received a burr hole cover, was re-operated 17 days after the primary operation for recurrent cSDH. No patient who had received a burr hole cover required a reoperation.

## Discussion

This study set out to explore the relationship between placement of a burr hole cover after burr hole trepanation for cSDH and patient satisfaction with the aesthetic result of the scar. In this small retrospective series, the patients rated the aesthetic results of the scar where the underlying burr hole had been covered by a plate (intervention group) more favorably than scars over uncovered burr holes. We also found that the rate of skin depressions was significantly lower over burr holes that had been covered by a plate and that there was no obvious increase in undesirable effects, such as wound pain or intra- or postoperative complications.

The results appear to be valid, as the actual observed outcomes compare well with the theoretically expected ones, conferred through the tested variable (placement of burr hole cover). Based on previous literature and our personal experience [7], it could be expected that if a group difference was observed, this difference would be in favor of the intervention group and measurable on the applied outcome metrics. Credibility of our findings is furthermore substantiated by internal consistency between the outcome measures (aesthetic satisfaction and rate of skin depression).

### Efficacy of burr hole covers to reduce scalp depression and improve aesthetic outcome

The research question addresses a frequent problem faced in daily clinical patient care—the undesirable long-term sequelae after burr hole trepanation, namely skin depression and unsatisfactory aesthetic outcome. This is especially the case in bald patients or those with sparse scalp hair. As cSDH have a male-to-female ratio of approximately 3:1 [5, 12], the problem of externally visible scalp depressions potentially applies to a great proportion of all cSDH patients. But even in patients with full hair, where scalp depressions may not be visible to others, they may interfere with ADLs, such as hair dressing or combing, in more than half of the patients [7].

**Fig. 5:**
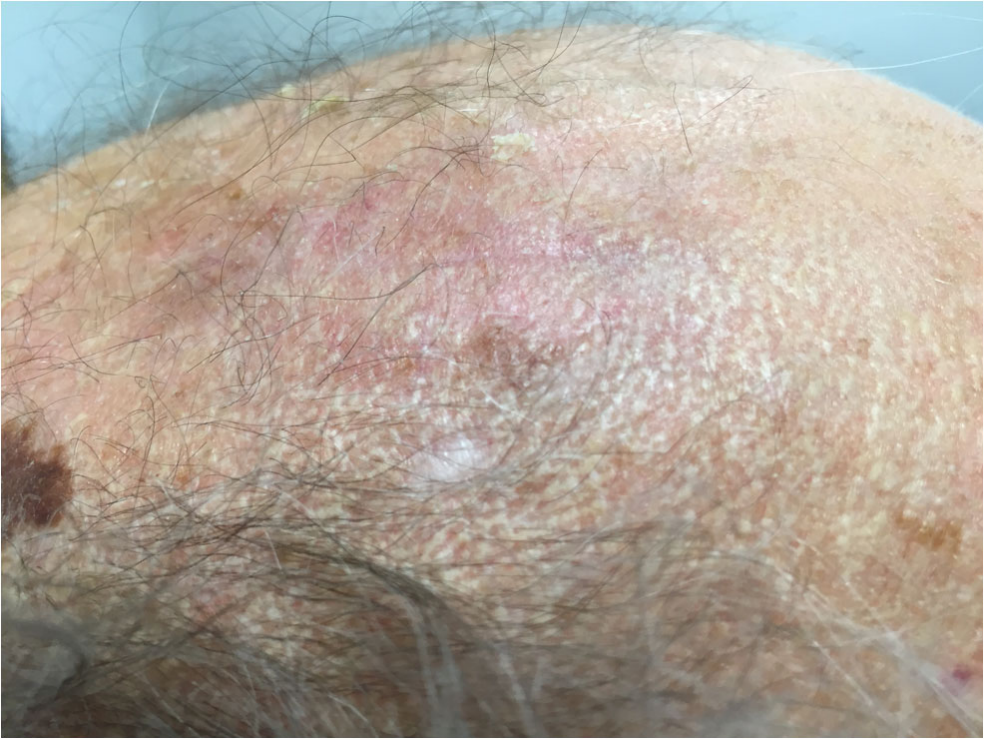
Illustrative picture of an 91-year-old male patient, seen in outpatient clinics about 16 weeks after right-sided frontal and parietal burr hole trepanation for a cSDH, with the trepanation site covered by a burr hole plate

Despite the high prevalence, we were surprised to learn that there was a scarcity of literature on this topic. Our results are consistent with the only available previous report on this topic, originating from South Korea [7]. In this retrospective cohort study, the authors measured significantly less (7.4 vs. 91.9%, *p* < 0.001) and smaller (0.16 vs. 2.45 mm, *p* < 0.001) scalp depressions on the computed tomography (CT) scans obtained in the follow-up period of patients after burr hole trepanation with or without placement of titanium burr hole covers. The authors also performed a telephone interview to ask patients with scalp depressions about their perceived impairment: about 74% reported having cosmetic complexes and 62% functional handicaps in the ADLs [7]. Our present findings are in line with the previous report, despite differences in the methodological approach. In our study, we applied a previously established scale on aesthetic satisfaction with the surgical site [4] and we asked patients to judge themselves whether or not the skin over the burr hole was depressed. Notwithstanding the methodological differences, both studies show an impressive reduction of skin depressions from > 90% over uncovered to < 10% over covered burr holes (Fig. 5). While the prior report did not assess patient-reported satisfaction—arguably the most important outcome—our present study indicates that the subjectively perceived aesthetical result is improved after placement of a burr hole cover. The improvement in patient satisfaction is likely conferred through the decreased prevalence of skin depressions, as wound pain was similarly low in the study and control group.

### Why is the placement of burr hole covers no standard of care?

In theory, burr hole covers represent an effective, easy-to-apply, and relatively inexpensive solution to prevent cosmetically and functionally unfavorable skin depressions. This adjunct to the regular surgical therapy has not yet found entry into clinical practice as standard of care, however. A variety of reasons may be responsible for this, including the lack of awareness by neurosurgeons, fear of increased intra- and postoperative complications, or the associated costs.

Awareness of treating physicians may be low due to the fact that skin depressions are not associated with an imminent risk to patient health. However, a patient’s perception of the cosmetic result is an important determinant of satisfaction with treatment. Owing to the demographic development and improvements in health care [8, 11], the proportion of elderly cSDH patients with long-term active participation in life after burr hole trepanation is increasing. Today’s patients have evolved into informed customers and service partners, no longer expecting only the performance of a basic surgical procedure but the delivery of favorable treatment outcomes topped with an excellent service [14].

Besides allergic reactions (extremely rare against titanium) [6], there is the potential danger of screws falling into the subdural space, which could prevent neurosurgeons from applying burr hole covers for this indication. For the material used in our department, screws do not fit between the gaps in the permeable burr hole covers. Therefore, by completely covering the burr hole and firmly pressing it onto the skull before receiving the screws from the scrub nurse, the risk of screw misplacement can be minimized.

The most important intervention- and disease-specific complication is cSDH recurrence, necessitating a repeated surgical intervention in 10–20% of cases [8, 9, 12]. Subsequently, there could be concerns as to whether the placement of burr hole covers interferes with hematoma reabsorption or drain placement. We preferably place subperiosteal drains, and the burr hole plates used in our department are permeable to allow for hematoma clearance from the subdural into the subperiosteal space. In the present series, no patient treated with a burr hole cover required a second surgical treatment for hematoma recurrence over a mean follow-up interval of 5 months. In the study by Im et al., drains were placed into the subdural space, and burr hole plates with a small gap were applied to account for the drain (Synthes GmbH, Oberdorf, Switzerland) [7]. In their series, the authors unfortunately do not report on hematoma recurrence or need for revision surgery.

They do, however, report on SSI that was *n* = 2/196 (1.02%) in their series, encompassing *n* = 96 patients with burr hole covers. Both cases of SSI occurred in patients that had not received a burr hole cover. Also taking into consideration that our present analysis did not identify a patient with SSI after placement of a titanium burr hole cover, the current literature suggests no obvious increase in infectious complications.

Both the previous group and ours did not experience any instrument failures such as screw loosening, burr hole cover displacement, implant protrusion, or scalp perforation [7].

Evidently, the application of osteosynthetic material results in higher treatment costs, and currently, there is no study that has addressed the question whether the perceived beneficial effect (aesthetic and functional) is worth the additional expenses. This is a difficult issue to study, as it is largely influenced by socioeconomic circumstances, and costs for insured patients emerge for the hospital or the health insurance company rather than the individual patient. In our practice, the additional costs for placement of two burr hole plates and four screws in a patient with unilateral cSDH amount to about SFr 260, Swiss francs (about EUR 225/USD 260). Surveying patients, neurosurgeons, and health insurance providers may prove useful in determining the perceived value of this intervention.

### Strengths and limitations

This is the first study to compare subjective patient satisfaction with the aesthetical result of the scar with or without placement of burr hole covers after trepanation for cSDH. Despite its small sample size, the study finds a significant difference on a previously established scale for aesthetic satisfaction.

Limitations include its retrospective nature, as well as potential bias by the selection of patients with and without burr hole covers. As patients were often aware of the fact whether or not a burr hole cover had been placed, subjects were not “blinded” at the time of the outcome evaluation. In addition, the study was underpowered to detect significant group differences for events with a low incidence, such as SSI or cSDH recurrences.

As for now, this retrospective pilot study reports innovative and promising findings. Therefore, a sufficiently large prospective, single-blinded randomized trial is currently developed in order to investigate whether the addition of a burr hole cover results in superior aesthetic outcomes, without an increase in the risks for complications (COveRs to impRove EsthetiC ouTcome after Surgery for Chronic subdural hemAtoma by buRr hole trepanation; CORRECT-SCAR trial).

## Appendix

Further contributors of the CORRECT-SCAR study group are listed in alphabetical order.

- David Bellut, MD
- Sandra Dias, MD
- Giuseppe Esposito, MD
- Jorn Fierstra, MD/PhD
- Dilek Könü-Leblebicioglu, MD
- Niklaus Krayenbühl, MD
- Nicolai Maldaner, MD
- Marian C. Neidert, MD
- Markus Oertel, MD
- Carlo Serra, MD
- Lennart H. Stieglitz, MD
- Julia Velz, MD
- Jan-Karl Burkhardt, MD

## Abbreviations and acronyms

ADL: Activities of daily life
ANA: Aesthetic numeric analogue scale
cSDH: Chronic subdural hematoma
CT: Computed tomography
NRS: Numeric rating scale
SD: Standard deviation
SSI: Surgical site infection
